# Additive Manufacturing of Resected Oral and Oropharyngeal Tissue: A Pilot Study

**DOI:** 10.3390/ijerph18030911

**Published:** 2021-01-21

**Authors:** Alexandria L. Irace, Anne Koivuholma, Eero Huotilainen, Jaana Hagström, Katri Aro, Mika Salmi, Antti Markkola, Heli Sistonen, Timo Atula, Antti A. Mäkitie

**Affiliations:** 1 Department of Otorhinolaryngology–Head and Neck Surgery, HUS Helsinki University Hospital, University of Helsinki, P.O.Box 263, FI-00029 HUS Helsinki, Finland; ali2116@cumc.columbia.edu (A.L.I.); anne.koivuholma@helsinki.fi (A.K.); katri.aro@hus.fi (K.A.); timo.atula@hus.fi (T.A.); 2 Vagelos College of Physicians and Surgeons, Columbia University, New York, NY 10032, USA; 3 Department of Mechanical Engineering, Aalto University, P.O.Box 14100, FI-00076 Aalto Espoo, Finland; eero.huotilainen@iki.fi (E.H.); mika.salmi@aalto.fi (M.S.); 4 Department of Pathology, HUS Helsinki University Hospital, University of Helsinki, P.O. Box 21, Haartmaninkatu 3, FI-00029 HUS Helsinki, Finland; jaana.hagstrom@hus.fi; 5 Department of Imaging, HUS Helsinki University Hospital, University of Helsinki, FI-00029 HUS Helsinki, Finland; antti.markkola@hus.fi (A.M.); heli.sistonen@hus.fi (H.S.); 6 Division of Ear, Nose and Throat Diseases, Department of Clinical Sciences, Intervention and Technology, Karolinska Institutet, Karolinska University Hospital, SE-17176 Stockholm, Sweden; 7 Research Program in Systems Oncology, Faculty of Medicine, University of Helsinki, FI-00014 Helsinki, Finland

**Keywords:** additive manufacturing, rapid manufacturing, stereolithography, 3D imaging, head and neck surgery, surgical oncology

## Abstract

Better visualization of tumor structure and orientation are needed in the postoperative setting. We aimed to assess the feasibility of a system in which oral and oropharyngeal tumors are resected, photographed, 3D modeled, and printed using additive manufacturing techniques. Three patients diagnosed with oral/oropharyngeal cancer were included. All patients underwent preoperative magnetic resonance imaging followed by resection. In the operating room (OR), the resected tissue block was photographed using a smartphone. Digital photos were imported into Agisoft Photoscan to produce a digital 3D model of the resected tissue. Physical models were then printed using binder jetting techniques. The aforementioned process was applied in pilot cases including carcinomas of the tongue and larynx. The number of photographs taken for each case ranged from 63 to 195. The printing time for the physical models ranged from 2 to 9 h, costs ranging from 25 to 141 EUR (28 to 161 USD). Digital photography may be used to additively manufacture models of resected oral/oropharyngeal tumors in an easy, accessible and efficient fashion. The model may be used in interdisciplinary discussion regarding postoperative care to improve understanding and collaboration, but further investigation in prospective studies is required.

## 1. Introduction

Additive manufacturing (AM), otherwise known as three-dimensional (3D) printing, is a growing technology with an emerging role in several surgical specialties. For example, it has been used for preoperative planning of hemihepatectomy and prostatectomy procedures [1,2,3]. Within otolaryngology, AM has improved surgical education and simulation, facilitated production of auricular prostheses and hearing aids, and enabled more accurate mandibular reconstruction following major segmental resection [4,5,6]. These are just a few examples of how AM may enhance patient-specific care, as there are several potential applications of this technology that have yet to be established. Postoperative management of head and neck cancer is one possible application that has not been well studied in the literature to date.

Clear visualization of head and neck tumors is essential not only for surgical planning but also postoperative management. Detailed preoperative imaging of the mass helps the surgeon achieve negative margins while mitigating surgical risks, including cosmetic deformity and neuromuscular injury. In the postoperative setting, tumor visualization is equally important. Head and neck surgeons must collaborate with pathologists, radiation and medical oncologists, and imaging specialists to evaluate the surgical margins, plan for possible revision procedures or adjuvant therapy, and monitor for recurrence. Knowledge of the surface anatomy, tumor structure, orientation, and location of surgical margins is essential for these multidisciplinary discussions to be effective.

Although magnetic resonance imaging (MRI) is the gold standard imaging modality for head and neck cancer [7], 2D MRIs do not necessarily show oral and oropharyngeal tumors from the clinician’s perspective. For one, they may limit appreciation of complex borders and anatomy. In addition, MRI interpretation can be challenging for the untrained eye, particularly when there are artifacts due to inflammation and/or swallowing [8]. Software and tools to combine preoperative MRIs and postoperative information about biopsies, tumor orientation, and surgical margins are not yet considered part of standard clinical practice.

The 3D-printed models of resected tumor tissue may improve visualization and understanding of tumor structure, location, and orientation in the postoperative setting. Previous studies have constructed 3D tumor models based on preoperative radiologic images, most commonly computer tomography (CT) and MRI scans [9,10,11], or histological reconstruction [12,13]. The 3D reconstructions of bone can also be made by using cone beam computed tomography [14,15]. However, it has previously been shown that standard digital photography may also be used to construct 3D models of human tissue [16]. This technique obviates the need for 3D CT/MRI or a 3D scanner, which may make digital photography the preferred modality for AM in certain settings. In addition, digital photography is very cost effective, accessible, and popular throughout much of the world. To our knowledge, no studies have assessed the feasibility of creating 3D tumor models using standard digital photographs of resected tumor tissue.

The objective of this pilot study is to determine whether digital photography be used to construct 3D images and physical models of resected oral and oropharyngeal tumor tissue. This study will therefore develop a system in which the tumors are resected, photographed in the operating room, and modeled/printed using AM techniques. Specific outcomes to be measured include the number of photographs required for modeling, production times, and costs. The 3D model of the resected tissue may serve as a communication tool at multidisciplinary tumor board meetings to facilitate discussion of critical surgical margins, tumor location, and orientation, which affects decision-making regarding postoperative care.

## 2. Materials and Methods

### 2.1. Participants

The study was approved by the institutional review board at HUS Helsinki University Hospital (Helsinki, Finland). Patients were eligible for inclusion in the study if they were diagnosed with an oral or oropharyngeal tumor and were scheduled for surgical resection. Three patients were identified through review of the operative schedule. Eligible patients underwent informed consent procedures and signed written consent forms to participate in the study. Hospital records of all patients included in the study were reviewed for clinicopathological data.

### 2.2. Clinical Care

All patients underwent a preoperative MRI of the head and neck. MRIs were performed at 1.5 T, with slice thickness of 3–4 mm. The imaging protocol was a standard protocol used for head and neck tumors. The mass was visualized and resected via partial resection of the tongue base (Case 1), hemiglossectomy (Case 2), or total laryngectomy with extension to the base of tongue (Case 3). Surgical resection was planned and performed according to standard protocol.

Immediately after resection, the resection block was placed on a white surgical towel in the operating room. The tissue was placed such that any visible area believed to be tumor was exposed to the viewer (i.e., not resting on the towel). The tissue was photographed by a study assistant using a smartphone (iPhone 6s; Apple; Cupertino, CA, USA) in natural light without flash. Photographs were taken at a distance approximately 15–30 cm away from the specimen at numerous angles. Every surface of the specimen was photographed from several different angles to aid in image alignment for 3D reconstruction. Attempts were made to image every surface of the tissue except for the inferior surface resting on the towel because movement of the specimen to photograph this region would have disrupted the tissue and resulted in inaccurate modeling. Approximately sixty to two hundred photographs were taken from several angles and planes with respect to the specimen. Variation in the number of photographs taken served to not only ensure that complex surface topography was sufficiently captured (with more complex topography requiring more photographs), but also to get a sense of how the number of photographs affected 3D image construction. Photographs did not contain any patient information. After all photographs were taken, the resection block was transferred to the Department of Pathology for histological analysis on the day of surgery.

### 2.3. Additive Manufacturing

Digital photos of the specimen were then imported into Agisoft Photoscan (version 1.4.2.6205; Agisoft; St. Petersburg, Russia), a 3D imaging software that performs photogrammetric reconstruction using a series of digital images. The software automatically aligned images using the “Highest” alignment accuracy settings and adaptive camera model fitting; non-aligned photographs were discarded from the project. After alignment, a dense point cloud was constructed with “Ultra high” quality, and “Moderate” depth filtering (boundary smoothing). Processing took place on a laptop workstation. Lowering the quality to the lowest available settings reduced the computation to a few minutes, but also reduced both the surface topology and texture (color) accuracy.

The dense point cloud was triangulated into a mesh using interpolation to fill any remaining holes, and the mesh was exported in a color-preserving PLY file format into Meshlab [17]. The table surface surrounding the section block was removed, and the hole below the model was stitched (no model surface was constructed on the undersurface because photographs were not taken from underneath the resection block). Finally, the model was converted into STL file format, which is the input supported by most 3D printers. Due to the STL standard lacking support for local color information, the model face colors were encoded into triangle normal values within the file.

In all cases colorized 3D models were printed using a binder jetting 3D printer (ProJet CJP 660, 3D Systems Inc. Rock Hill, SC, USA) from composite powder hardened in post processing with cyanoacrylate. In Case 1, a 3D model was also printed using material extrusion (uPrint SE Plus, Stratasys Ltd., Rehovot, Israel) from ABS Plus material and SR-30 Soluble Support Material.

**Figure 1:**
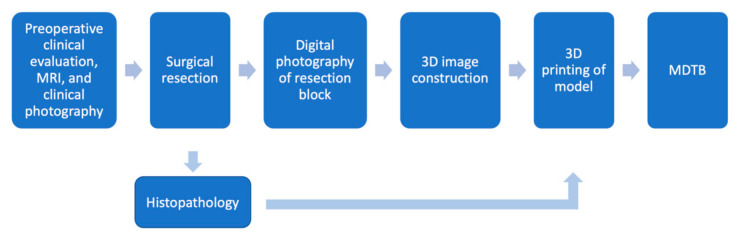
A diagram showing various steps of the project and their interaction (arrows), MDTB: multidisciplinary tumor board.

The different steps of the project and their relationships to one another are presented in Figure 1.

### 2.4. Multidisciplinary Tumor Board Meeting

Surgical outcomes were reviewed at a meeting of the multidisciplinary tumor board (MDTB) two weeks after surgery. MDTB meetings consist of case review and discussion among pathologists, head and neck surgeons, oncologists, radiologists, and other specialists. The need for revision surgery or adjuvant therapy was assessed for each case.

## 3. Results

### 3.1. Case 1

A 62-year-old female was diagnosed with an exophytic tumor on the left side of the tongue base. Preoperative clinical classification was T1N0M0. Resection of the tumor was performed with selective neck dissection of levels IIa, IIb, and III. Approximately two weeks later, the case was discussed at an MDTB meeting. Histological analysis confirmed a p16-negative pT2pN0, Stage II squamous cell carcinoma (SCC) with a diameter of 30 mm. Maximum invasion was 4.5 mm and the closest surgical margin was 4 mm at the posterior border. Due to the extent of invasion, postoperative radiation was recommended as the next step in the patient’s care.

### 3.2. Case 2

**Figure 2:**
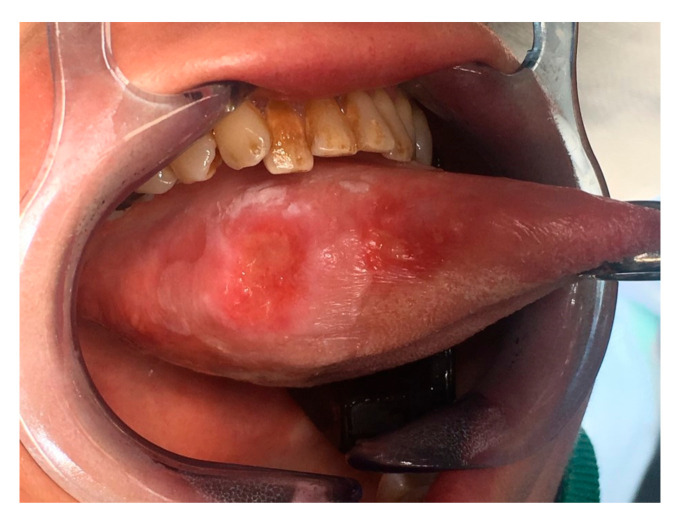
Preoperative image of Case 2 showing squamous cell carcinoma of the right mobile tongue.

**Figure 3:**
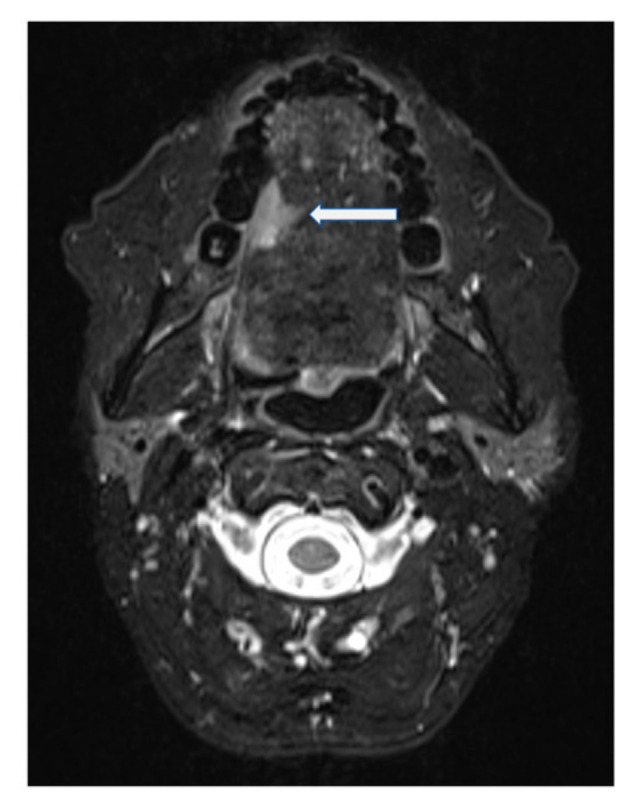
T2-weighted fat-suppressed, contrast-enhanced MRI of Case 2 in the axial plane. Squamous cell carcinoma indicated by arrow.

A 53-year-old female was diagnosed with a p16-negative T3N2bM0, Stage IVa SCC of the mobile tongue at an outside institution (Figure 2 and Figure 3). Hemiglossectomy was performed in conjunction with neck dissection of levels I, IIa, III, and IV on the right side. Anterolateral thigh microvascular flap was utilized in reconstruction. Histopathology revealed an SCC with a diameter of 24 mm and with 10 mm depth of invasion. The minimum resection margin was 7 mm on the medial edge of the tumor. She had two metastatic lymph nodes in level IIa with no extranodal extension. The pathological staging was pT2pN2bM0 (Stage IVa). MDTB recommended postoperative chemoradiotherapy.

### 3.3. Case 3

A 52-year-old male, ex-smoker, was diagnosed with SCC of the vallecula. Seven years prior, the patient had been diagnosed with SCC (pT2pN2bM0) in the floor of the mouth and SCC in the esophagus. Both oral and esophageal carcinomas had been treated with surgery and chemoradiotherapy. Due to residual tumor in the esophagus, the patient had undergone surgical resection with gastric pull-up.

In 2018, the patient presented with swallowing difficulty. A third primary tumor in the vallecula was diagnosed clinically as a p16-negative T2N2bM0 (Stage IVa) SCC with dimensions 15 × 5 × 35 mm on MRI scans. There were metastatic lymph nodes on the left side of the neck. The patient underwent total laryngectomy with extension to the base of tongue and neck dissection on the left side including levels II to V. Frozen sections revealed that the initial resection was inadequate. Further resection was performed during the same operation and repeated frozen sections as well as final pathology displayed that the margins were free of tumor. Final histopathology also revealed nerve and blood vessel invasion. The tumor was classified pT2pN2bM0.

### 3.4. Digital Reconstruction and Additive Manufacturing Process

**Figure 4:**
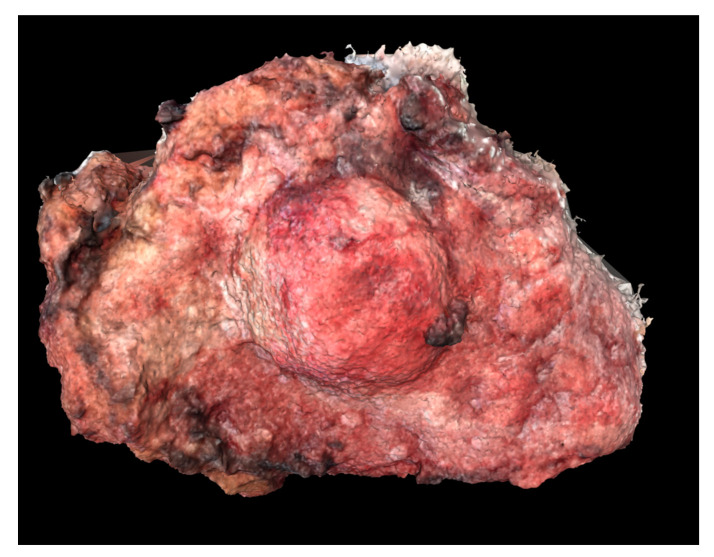
Digital 3D model of Case 1 using Meshlab.

**Figure 5:**
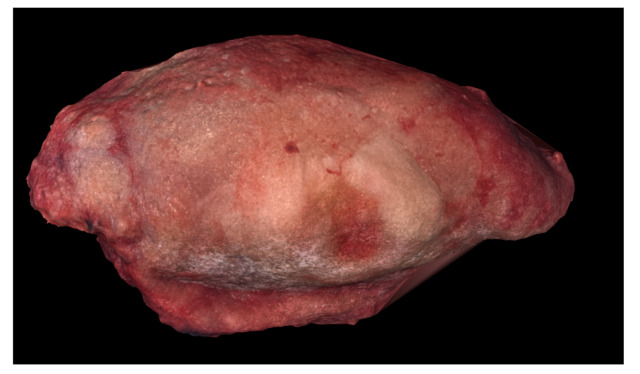
Digital 3D model of Case 2 using Meshlab.

**Figure 6:**
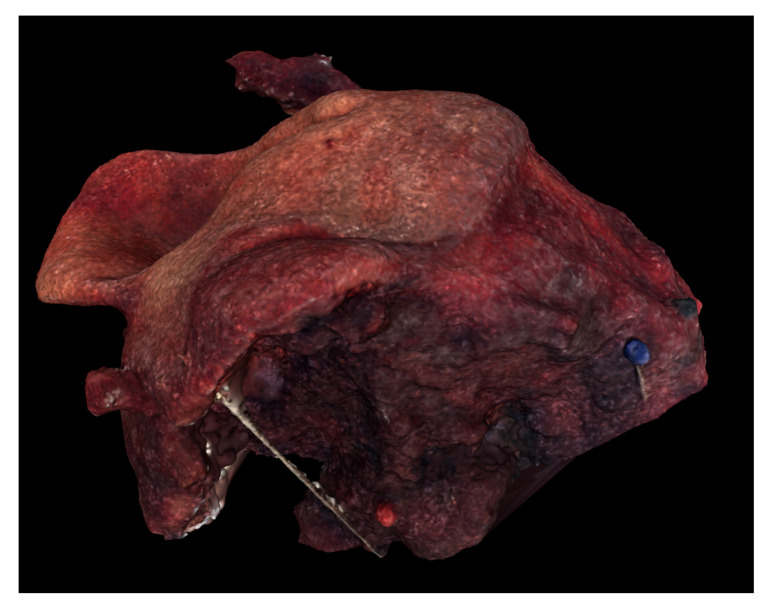
Digital 3D model of Case 3 using Meshlab.

Table 1 summarizes the main parameters from the reconstruction. “Photographs taken” refers to the number of smartphone photographs, taken from various orientations, introduced into the photogrammetry software. Aligned photographs were able to be utilized during the computation and construction of the digital 3D model (Figure 4, Figure 5 and Figure 6). Processing time gives an approximate total time span required by the alignment, dense cloud creation, and meshing steps.

**Figure 7:**
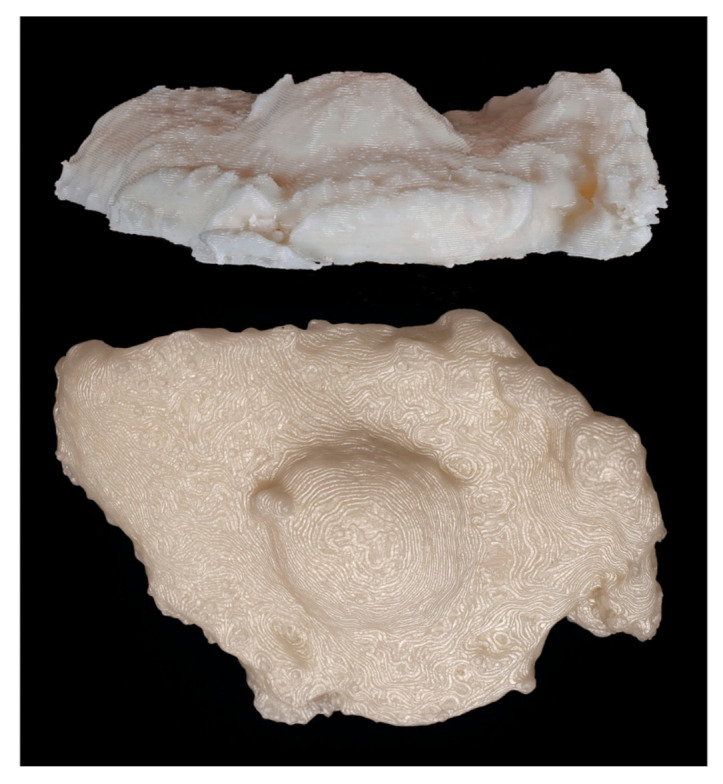
Lateral and superior views of physical 3D model of Case 1 printed via material extrusion.

**Figure 8:**
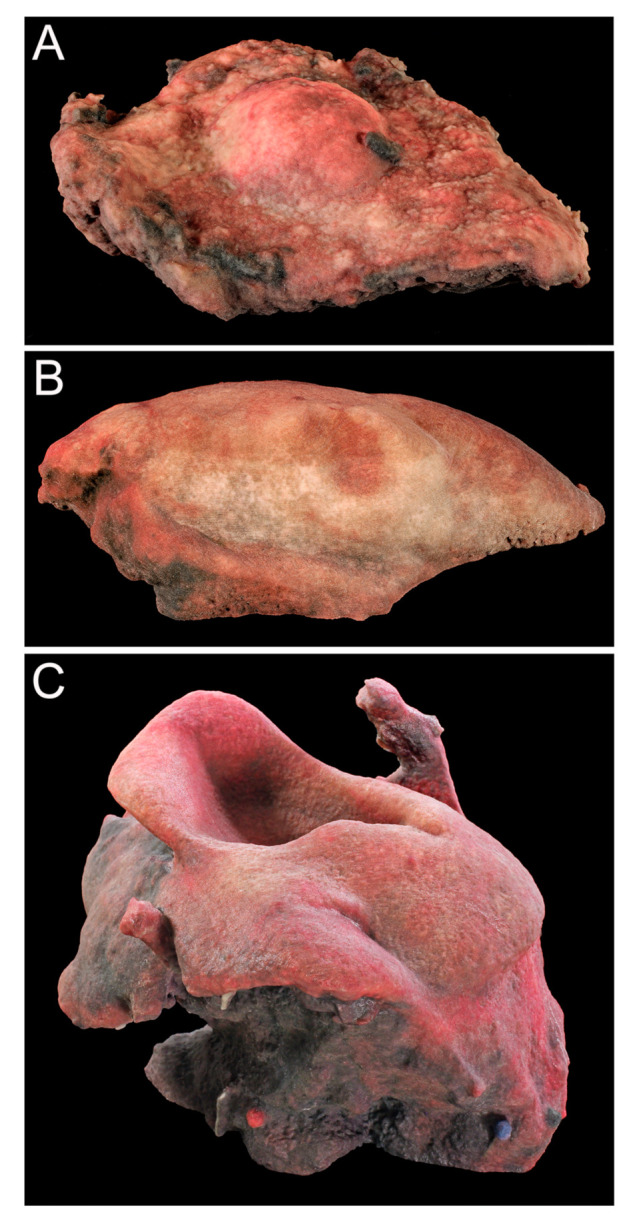
Physical 3D models of Case 1 (**A**), Case 2 (**B**), and Case 3 (**C**) printed via binder jetting.

Digital and physical models of the surgical resection block were produced within one week of the operation. The 3D model dimensions were scaled up from the true dimensions of the resection block due to the small size of each tumor. Enlarging the dimensions of the model allowed for improved visualization of surface topography and anatomical details. For Case 1, the model produced by binder jetting conveyed more information than the material extrusion model because of the added color texture made possible by binder jetting (Figure 7 and Figure 8). Using material extrusion, the model was made of simple ABS-like white polymer with less detail.

Production time for printing of the 3D model ranged from two to nine hours and printing costs ranged from 25 to 140 EUR: 24.90 EUR/28.41 USD (Case 1), 38.20 EUR/43.59 USD (Case 2) and 141.00 EUR/160.89 USD (Case 3).

## 4. Discussion

In this pilot study, three surgical resection blocks of oral and oropharyngeal cancer were photographed, modeled, and printed using AM technology. Each tumor originated in a different anatomical location: the mobile tongue, base of tongue, and epiglottic vallecula. This study employed digital photography to construct a 3D model of each resection block. Processing time for 3D image construction ranged from seven to 24 h and importing a larger number of photographs into Agisoft required longer processing times to construct the 3D image. Production time for printing of the 3D model ranged from two to nine hours and printing costs ranged from 25 to 141 EUR (28 to 161 USD), with larger models requiring more material consumption, longer printing times, and higher costs. Overall, the process was relatively efficient and could feasibly be completed within one day depending on the number of photographs and size/complexity of the tissue specimen.

There was also variability in the number of photographs processed for each specimen. For Case 3, 195 photographs of the resection block were taken, partly because of the sample’s convoluted shape with its cavity structure. This high number of photos increased processing time but did not add to the quality of the digital or printed model. In practice, models of satisfactory quality can easily be reconstructed using a magnitude fewer number of photographs, which also reduces processing time, as long as all orientations of the specimen are captured allowing the software to accurately align the photographs.

The oral cavity and oropharynx are challenging surgical environments. Many procedures in these regions are restricted to small operative areas, involve complex anatomy, and put the patient at risk for cosmetic and functional defects. There are few landmarks to aid in surgical resection apart from the free mucosal border, as the deep margin is typically surrounded by muscle and connective tissue. Moreover, attempts to achieve adequate surgical margins must be balanced with tissue preservation to not only avoid neighboring structures, but also maintain speech and swallowing functions [18]. At our institution, at least 5 mm microscopic margins are warranted for carcinomas in the oral cavity and oropharynx. Inadequacy or invasion of the margin is known to at least double the probability of local recurrence [19,20,21,22]. Head and neck surgeons, pathologists, radiologists, oncologists, and other specialists must collaborate to review the adequacy of margins and assess the need for revision surgery or adjuvant therapy. However, information exchange across disciplines can be imprecise or incomplete when referring only to imaging studies and histopathology slides. If the margin shown on a histopathology slide is positive or insufficient, it can be challenging to correlate that area on the slide with the exact location in the resected tissue.

Diagrams drawn by the pathologist depicting how the tissue was cut during slide preparation may prove useful when the tumor is small and simple in shape but may lack specificity and accuracy in more complex cases. Referring to the preoperative MRI is another option. MRI has superior soft tissue contrast resolution and the relationship of the tumor to nearby structures can usually be sufficiently evaluated. However, correlation of the MR findings and histopathological information about the orientation and positive margins of the tumor is usually difficult to determine precisely. The use of 3D modeling to correlate MR data to histopathological findings has previously been studied primarily in cases of prostate cancer [23,24,25,26], but these techniques have not been used in routine clinical practice. Lastly, from a clinical perspective, postoperative swelling, hematoma, or reconstructive procedures can distort the appearance of the resection defect and make it difficult to identify areas requiring additional treatment.

Because of these limitations, more effective tools are needed to facilitate interdisciplinary communication. Image-guided surgical navigation is one example. Using preoperative 3D images (CT or MRI) and a real-time imaging source connected to input devices, the surgeon can better understand the orientation, position, size, and location of a tumor in relation to other structures. Guijarro-Martinez et al. demonstrated how image-guided navigation may be used during head and neck tumor surgery, focusing on the importance of mapping resection margins, the locations of any intraoperative biopsies, and any areas suspicious for residual tumor [18]. During the procedure, they saved digitized coordinates of these areas on an intraoperative CT scan. Pathologists accessed this anatomical map to locate any tumor-positive coordinates in relation to the resection margins. This map also allowed radiotherapists to focus precisely on tumor-positive coordinates identified by the pathologist, thereby avoiding irradiation of adjacent healthy tissue.

As image-guided navigation technology continues to emerge in head and neck surgery, we believe that AM can serve a similar purpose. Our objective was to develop a simple, replicable process of 3D modeling and printing resected oral tongue and tongue base tumors. These models would then be used at MDTB meetings to facilitate interdisciplinary management (Figure 1). Digital photography enabled us to efficiently construct a 3D image of the resection block, which was then printed to create a tangible model of the tissue. Like the coordinate map used in image-guided surgical navigation, this model optimizes visualization and understanding of tumor topography, location, and orientation. For example, pathologists could use the model as a tool to present exact margin locations. While postoperative 3D tumor modeling has not been well studied in head and neck literature, we believe it merits further investigation given the efficiency of the process (as short as 1 or 2 days from surgery to printing), growing availability of AM technology and related open-source software, and developing need for non-invasive procedures that mitigate surgical risks. Future directions in developing our method may include using a 3D scanner instead of photography and comparing the accuracy of different methods. While photography is inexpensive and widely available, 3D table scanners have become more advanced and accessible and have been used in 3D soft tissue tumor modeling [27]. Using a scanner may affect the speed and accuracy of the 3D modeling process. 

Apart from optimizing interdisciplinary communication and postoperative care, 3D tumor models have multiple applications that have been demonstrated in several areas of the body. For example, Hovens et al. recently studied postoperative 3D modeling of prostate tumors to understand the relationship between tumor morphometry and prognosis [12]. 3D modeling has also been shown to facilitate preoperative planning for malignant bone tumors in the cervical spine, complex cardiac and thoracic malignancies, and hilar cholangiocarcinoma [28,29,30,31]. Moreover, 3D imaging, 3D modeling and AM have also played a role in mathematical optimization of resection planes and allograft fitting, as previously demonstrated in fresh cadaveric osteochondral allograft knee surgery [32]. Similar studies can be conducted using 3D models of head and neck tumors. Currently AM and 3D technologies are utilized mostly in medical models, guides and implants [33] and not as frequently in surgical settings focusing on tissue resection. As 3D printing technology becomes more simplified and available to academic medical centers and hospitals on a global scale, we expect the clinical and research applications to greatly expand.

Despite the potential of this technology, there are limitations that must be recognized when determining how and when it should be utilized. As with any equipment, mechanical issues are to be expected, and contingency plans should be considered if 3D printing is routinely incorporated into patient care. In addition, using digital photography to construct the 3D image may limit visualization of the aspect of the tissue resting on the underlying surface. In our study, the specimens were not rotated to photograph all aspects because of the risk of disturbing the tissue’s shape, which would interfere with image alignment in the 3D modeling process. In the future, set-up for photographing the resection block could be improved by developing a stand or a rack that would allow the entire surface area to be captured. Other potential limitations include the printing costs, such as the cost of the printer itself, maintenance of the printer, printing materials, operating staff, and so on. 

## 5. Conclusions

This 3D imaging and printing technology can be utilized in the management of head and neck tumors in a way that is easily implemented, accessible, and efficient. A physical model of the oral/oropharyngeal cancer resection block can be used to improve understanding of tumor orientation and optimize interdisciplinary collaboration. Certain limitations, including mechanical error, surface visibility, and cost, are areas for future development. Larger prospective studies are required to further investigate the role of additive manufacturing in head and neck surgery and establish recommendations on how to best implement this technology.

## Figures and Tables

**Table 1:** Stereophotogrammetry reconstruction details using binder jetting 3D printer.

Case	Photographs Aligned/Total	Stereophotogrammetry Processing Time (h)	Scaling Factor	Print Time (h:min)	Print Volume/Material Consumption (cm^3^)
1	46/63	7	2.5	2:01	139.92
2	111/117	16	2.0	3:39	227.63
3	194/195	24	1.5	9:09	976.82

## Data Availability

Data sharing is not applicable to this article.
